# Italian regional health service costs for diagnosis and 1-year treatment of ADHD in children and adolescents

**DOI:** 10.1186/s13033-017-0140-8

**Published:** 2017-04-28

**Authors:** Gianluigi Casadei, Massimo Cartabia, Laura Reale, Maria Antonella Costantino, Maurizio Bonati, for the Lombardy ADHD Group, Stefano Conte, Valeria Renzetti, Laura Salvoni, Massimo Molteni, Sara Trabattoni, Paola Effedri, Elena Filippini, Elisabetta Pedercini, Edda Zanetti, Nadia Fteita, Daniele Arisi, Roberta Mapelli, Simona Frassica, Simonetta Oriani, Christian Trevisan, Susanna Acquistapace, Ottaviano Martinelli, Davide Villani, Emanuela Binaghi, Andrea Deriu, Ernesta Ricotta, Arianna Borchia, Paola Morosini, Maddalena Breviglieri, Giuseppe Capovilla, Roberto Segala, Claudio Bissoli, Maria Paola Canevini, Antonella Costantino, Isabella Cropanese, Anna Didoni, Emiddio Fornaro, Silvia Merati, Alberto Ottolini, Monica Saccani, Roberto Vaccari, Vera Valenti, Alessandra Valentino, Umberto Balottin, Matteo Chiappedi, Elena Vlacos, Corrado Meraviglia, Maria Grazia Palmieri, Gianpaolo Ruffoni, Francesco Rinaldi, Federica Soardi, Chiara Luoni, Francesca Pavone, Giorgio Rossi, Maurizio Bonati, Massimo Cartabia, Laura Reale, Michele Zanetti

**Affiliations:** 1 CESAV-“Angelo e Angela Valenti” Centre for Health Economics, Department of Public Health, IRCCS-Istituto di Ricerche Farmacologiche “Mario Negri”, Via Giuseppe La Masa, 19, 20156 Milan, Italy; 2 Laboratory for Mother and Child Health, Department of Public Health, IRCCS-Istituto di Ricerche Farmacologiche “Mario Negri”, Milan, Italy; 3 Child and Adolescent Neuropsychiatric Unit, IRCCS Foundation Ca’ Granda, Ospedale Maggiore Policlinico, Milan, Italy

**Keywords:** Health care costs, Child and adolescent health, Mental health, Ambulatory/outpatient care

## Abstract

The main aim of this study was to estimate the costs associated with diagnostic assessment and 1-year therapy in children and adolescents enrolled in 18 ADHD reference centres. Data concerning 1887 children and adolescents from the mandatory ADHD registry database during the 2012–2014 period were analysed. The overall diagnostic and treatment costs per patient amounts to €574 and €830, respectively. The ADHD centre, the school as sender, and the time to diagnosis constitute cost drivers. Non-pharmacological therapy resulted as being more expensive for patients concomitantly treated with drugs (€929) compared to those treated with psychological interventions alone (€590; p = 0.006). This study gives the first and reliable estimate of the costs associated with both diagnosis and treatment of ADHD in Italy. Although costs associated with mental disorders are difficult to estimate, continuing efforts are need to define costs and resources to guarantee appropriate care, also for ADHD.

**Electronic supplementary material:**

The online version of this article (doi:10.1186/s13033-017-0140-8) contains supplementary material, which is available to authorized users.

## Background

The costs of psychiatric disorders have been scantly investigated, particularly with regard to those that affect children and adolescents [1, 2]. Methodological complexities justified the lack of comprehensive estimates of the economic impact associated with the burden of psychiatric disorders [3, 4]. World-wide up to 20% of children and adolescents suffer from a neuro-psychiatric condition; in developed countries, both mental and neurological disorders account for the 40% of the burden of all brain diseases and for the 35% in Europe only [5–8].

Attention deficit hyperactivity disorder (ADHD) is a neurobiological disorder [9] characterized mainly by clinical manifestations such as difficulty in paying attention, impulsive behaviour, and a heightened level of physical activity, occurring more frequently and intensely than in other children of the same age or developmental level [10]. ADHD symptoms usually become more evident in school aged children, are more frequent in boys than girls (ratio 3:1), and tend to persist into adulthood [11]. ADHD accounts as the third most common mental disorders in children and adolescents [12].

Despite a pooled worldwide ADHD prevalence in children and adolescents of 5.3%, there is wide variability between and within countries [13]. Such variability in prevalence rates may be explained by the different methodologies, diagnostic procedures, and criteria used in the studies [14, 15] as well as by the different settings and cultural approaches considered [16, 17]. However, when standardized diagnostic and impairment assessment procedures are followed, prevalence does not seem to have changed over time nor to have differed in the geographic locations considered [18]. According to national and international guidelines [19–22], ADHD treatment should be based on a multimodal approach combining psychosocial interventions with pharmacological therapies, and should take into consideration the subject’s characteristics, including age, symptom severity, co-morbid disorders, cognitive level, and social and family context.

The impairments of ADHD are multi-faceted and occur in multiple settings, as costs associated with ADHD impact multiple societal costs within and outside the healthcare sector [23, 24]. Studies on the economic burden of ADHD within Italy, where healthcare is provided to all citizens, are currently lacking [25, 26]. An understanding of the costs associated with ADHD in children and adolescents is important for public policy makers as a rationale for improving and planning public services for both diagnosis and treatment [27].

Overall, in addition to the human suffering they cause, psychiatric disorders are among the most expensive of all health problems in adults [24, 28], even if evidence regarding the costs of psychiatric disorders has been slow to accumulate, particularly with regard to those that affect children and adolescents [29]. Literature review of the health care and treatment-related ADHD economic impact estimates an annual cost, specifically for children and adolescents, at $14,576 per individual, with a comprehensive range from $36 billion to $52.4 billion considering an ADHD prevalence rate of 5% [30]. However, these estimates are incomplete and inaccurate due to the fact that the majority of the existing studies did not fully assess all the potential costs related to an ADHD diagnosis [31]. A few reviews covering the attempts made at defining economic impact of ADHD by the limited number of available, country-based studies, highlighted a wide range in the magnitude of the societal cost estimates [30, 32–38].

In June 2011 an official ADHD regional registry was activated in the Lombardy Region, designed as a disease oriented registry collecting information on all subjects who access ADHD centres for a suspected ADHD diagnosis [39–41], as part of a regional project aimed to define a common approach to, and improve, ADHD diagnosis and therapy. In the Lombardy Region, during the study period, a network of 34 Child and Adolescent Neuropsychiatric Services (CANPS) provide care at the hospital (tier three) and community (tier two) levels for children and adolescents with neurologic, neuropsychologic and/or psychiatric disorders, and for their families. About 15% of the Italian pediatric population live in this region. Regional health authorities are responsible for the accreditation of the ADHD reference centers in regional hospitals (“ADHD centers”), as specialized ADHD hubs (tier three) of the CANPS network.

In such a context, the objective of this study was to estimate the costs, from the National Health Service (NHS) perspective, associated with diagnostic assessment and 1-year therapy in children and adolescents aged 5–17 years enrolled in any of the 18 ADHD reference centres of the Lombardy Region between January 2012 and December 2014 for suspected ADHD.

## Methods

This study was designed as a review of patient medical records identified from the Regional ADHD Registry database, to estimate the costs of the diagnostic assessment and 1-year therapy for subjects with a suspected ADHD diagnosis. The research was approved by the Institutional Review Board of the IRCCS—Istituto di Ricerche Farmacologiche “Mario Negri” Milan, Italy. Written informed consent was obtained for all patients to put information in the registry database and analyze them anonymously.

### Setting

This study is part of a specific project supported by the Regional Health Ministry and aimed to ensure appropriate ADHD management for every child and adolescent once the disorder is suspected and reported, and includes commonly acknowledged diagnostic and therapeutic procedures as well as educational initiatives for health care workers (child psychiatrists and psychologists) of the Lombardy Region’s health care system. The project’s participants are all the 18 ADHD centres of the Lombardy Region, the most economically important and populated Italian region with 1.690.127 citizens under 18 years old, and with an average income earned per person equal to € 24.005 in the study period. The ADHD centres, accredited by regional health authorities, are the hubs specialised in ADHD (Tier 3) of the Child and Adolescent Neuropsychiatric Services (CANPS) network and provide diagnosis and treatment care free, or at a nominal charge, working mainly on an outpatient basis and in close connection with educational and social services. ADHD centres are also responsible for the prescription of pharmacological therapies and their monitoring over time. Moreover, ADHD centres are responsible for inputting data into the official ADHD registry and for providing parent, teacher, and child training treatments [39–41].

### Study population and pathways of care

Anonymized, updated data of the official ADHD registry of the Lombardy Region (as of 31 August 2015) were available, with 3163 subjects enrolled in the June 2011–August 2015 period. The study population includes children and adolescents from January 2012 to December 2014 who had both a first outpatient visit at one of the 18 ADHD centres for a suspected ADHD diagnosis in the same period and had a complete diagnostic evaluation and treatment prescription at the time of data extraction. Our goal was to identify only children who had never been evaluated and treated before for ADHD. We further required that all of the study children with a confirmed ADHD diagnosis were received a care continuity at the ADHD centre within a 1-year period.

The guideline for all clinicians at the ADHD centres is to use the Diagnostic and Statistical Manual of Mental Disorders, Fourth Edition [42] criteria for diagnosing ADHD. Moreover, to define an optimal, evidence-based, shared strategy for diagnostic evaluation, an ad hoc assessment working group was created, involving a child neuropsychiatrist and a psychologist from each participating ADHD center and a group of researchers of the registry coordinating center (IRCCS—Istituto di Ricerche Farmacologiche “Mario Negri”). More specifically, also according to the recommendations of the Italian guidelines [22, 43], this strategy consisted of seven mandatory steps to be applied at the time of diagnostic evaluation: (1) a clinical anamnestic and psychiatric interview; (2) the neurological examination; (3) the evaluation of cognitive level by Wechsler Scales [44]; (4) the Schedule for Affective Disorders and Schizophrenia for School-Age Children (K-SADS) [45] for a complete psychopathology overview and comorbidity assessment; (5) the Child Behavior Checklist (CBCL) and/or the Conners’ Parent Rating Scale–Revised (CPRS-R) rated by parents; (6) the Conners’ Teacher Rating Scale–Revised (CTRS-R) rated by teachers; [46–48] and (7) the Clinical Global Impressions-Severity scale (CGI-S) [49] to quantify symptom severity. This diagnostic pathway was agreed on, approved, and shared by all participating ADHD centers.

Once a patient receives a diagnosis of ADHD and a treatment prescription, the registry is designed to provide differently structured types of follow-up visits at periodic intervals: at 3 and 6 months after the diagnosis, and every 6 months afterward for all patients; while for those given methylphenidate at 1 week and 1 month after the diagnosis also (after only 1 month if they received atomoxetine or other psychotropic drugs). Moreover, patients that receive a methylphenidate prescription need to perform a visit called “dose-test” which is carried out in day hospital regimen before starting the drug treatment.

### Data analytic procedures

Complete data of all eligible patients were extracted. Besides anamnestic and clinical information regarding age, gender, diagnosis and comorbidity, detailed information was available on a patient medical records basis for the following cost domains:

- Diagnostic pathway: all services supplied to patients with a suspected ADHD diagnosis, whether or not confirmed, by an agreed and shared child and adolescent neuropsychiatrists’ and psychologists’ assessment. In addition, the working days elapsed from the request a diagnosis of ADHD were calculated. In line with the regional health service perspective, only direct healthcare costs were estimated. Unit costs related to diagnostic tests were derived from tariffs reimbursed by the Lombardy Region in 2014. Detailed information is presented in Additional file 1: Table S1.
- Non-pharmacological therapies: Prescribed by physicians specialised in child and adolescent neuropsychiatry working in one of the 18 ADHD centres and provided by other therapists for example psychologists, also working within the ADHD centre. The direct health cost of non-pharmacological therapies was assessed by multiplying the estimated number of annual visits per patient by the unit costs derived from tariffs reimbursed by the Lombardy Region in 2014. Detailed information is presented in Additional file 1: Table S2.
- Pharmacological treatments: Prescribed by medical doctors specialised in child and adolescent neuropsychiatry working in one of the 18 ADHD centres (only prescriptions filled by patients). Drug utilization data were also derived from the Regional ADHD registry [39, 40]. Detailed information is presented in Additional file 1: Table S3.

Data on the total annual consumption of ADHD medications that require a therapeutic plan prescription form were evaluated over 1 year after the date of the diagnosis. The trends in total consumption of ADHD drugs were also analysed based on changing patterns of the drugs’ prescription practices, dosages, and formulations.

The unit costs of prescribed drugs were assessed based on: (a) price to public of medicines fully reimbursed by the Italian NHS and supplied through retail pharmacies (class A); or (b) sum of ex-factory price and 10.2% distribution margin of fully reimbursed drugs under direct distribution. Patient co-payment was not considered as it impacted a minority (<10%) of patients under drug treatment. The cost per milligram was calculated on the basis of the prescribed formulation (immediate- or extended-release, tablet, capsule, drops) by selecting the generic, when marketed. Annual drug consumption was estimated for each patient by multiplying the daily dosage prescribed at each visit by the number days until the following visit.

Laboratory tests,—including blood count, blood sugar, haemoglobin, ferritin, albumin, bilirubin, transaminases, gamma-glutamyl transpeptidase, erythrocyte sedimentation rate, thyroid hormones, uric acid, BUN, creatinine, urine—and EKG were carried out in day hospital (DH) setting only in patients undergoing pharmacological treatments, according to the Italian guidelines [22]. Hence, they were considered as pharmacological treatment-related expenditures. A DH unit cost of €232.00 was derived from DRG code 431 reimbursed by the Lombardy Region in 2014. Hospitalization (inpatient regimen) costs were been estimated as no patient was hospitalized.

All data were analysed with SAS Version 9.2, (SAS Institute, Inc., Cary, NC, USA). Descriptive statistics were computed for the entire study population and for subgroups. As costs of diagnostic pathways (recommended, optional, and total), time to diagnosis, and treatment costs were not normally distributed, median and inter quartile ranges were analysed. The Student’s t test was used to compare continuous variables (baseline clinical and anamnestic data), while χ^2^ (baseline clinical and anamnestic data), Wilcoxon–Mann–Whitney (diagnosis related costs, time to diagnosis evaluation), and Kruskal–Wallis (treatment and diagnosis related costs) tests were used to compare categorical variables. A multivariate logistic regression analysis with stepwise selection was carried out to assess the socio-demographic (baseline personnel and familiar data) and clinical, service models’ characteristics (clinical and organizational determinants). Moreover, a multivariate linear regression analysis was performed to assess the drivers of diagnostic costs and time to diagnosis.

## Results

### Baseline characteristics

Data concerning 1887 children and adolescents who accessed 1 of the 18 ADHD centres for the first time during the 2011–2015 period and. completed the diagnostic evaluation at the time of data extraction (September 1, 2015; Table 1) were analysed. These patients had a median age of 9 years (range: 5–17 years) at their first visit, and 1597 (85%) were males and 290 (15%) were females. In all, 1276 patients (68%) met DSM-IV-TR criteria (43) for ADHD: 1099 (86%) males and 177 (14%) females. In all, 1189 of 1887 (63%) enrolled patients had one or more psychiatric disorders (346 without ADHD) (learning disorders, 56%; sleep disorders, 19%; anxiety and mood disorders, 19%; oppositional defiant disorder, 17%; other, 13%), whereas 163 (9%) had a concomitant chronic diseases (neurological, 31%; respiratory, 30%; gastrointestinal, 7%; other, 19%). As shown in Table 1, the main anamnestic characteristics significantly associated with ADHD diagnosis were: lower age, presence of support teacher (at the time of assessment), ADHD familiarity, and an associated psychiatric disorder.

**Table 1:** Demographic and clinical characteristics of the sample population

Characteristics	Total sample (*N* = 1887)	With ADHD (*n* = 1276)	Without ADHD (*n* = 611)	p*
Age *M* (*SD*); median	9.3 (2.5); 9	9.1 (2.4); 9	9.7 (2.5); 9	<0.0001
5–11, *n* (%)	1506 (80)	1038 (81)	468 (77)	0.0161
12–17, *n* (%)	381 (20)	238 (19)	143 (23)	
Male: female	1597:290	1099:177	498:113	0.0092
Only child, *n* (%)	453 (24)	328 (26)	125 (20)	0.0116
Born abroad, *n* (%)	99 (5)	76 (6)	23 (4)	0.0453
Adopted, *n* (%)	63 (3)	54 (4)	9 (1)	0.0018
School variables, *n* (%)				
Grade				
Primary	1417 (75)	980 (77)	437 (72)	0.0139
Middle	465 (25)	293 (23)	172 (28)	
Repeaters	99 (5)	61 (5)	38 (6)	ns
Support teacher	142 (8)	123 (10)	19 (3)	<0.0001
Parent/family variables, *n* (%)				
High school graduate				
Mother	1070 (64)	694 (63)	376 (66)	ns
Father	871 (53)	562 (52)	309 (55)	ns
Employed				
Mother	1235 (72)	840 (74)	395 (67)	0.0028
Father	1584 (95)	1046 (95)	538 (94)	ns
Family history of ADHD	375 (20)	293 (23)	82 (13)	<0.0001
Anamnestic data, *n* (%)				
Pregnancy				
Cesarean section	465 (26)	333 (28)	132 (23)	0.0207
Preterm (<37 weeks)	168 (9)	120 (10)	48 (8)	ns
Low weight (<2500 g)	149 (9)	107 (9)	42 (7)	ns
Motor delay	85 (5)	60 (5)	25 (4)	ns
Language delay	366 (20)	266 (22)	100 (17)	0.0167
Psychiatric disorders, *n* (%)				
One or more	1189 (63)	843 (66)	346 (57)	<0.0001
Other chronic medical conditions, *n* (%)				
One or more	163 (9)	110 (9)	53 (9)	ns

* t test for continuous and χ^2^ for categorical variables

### Diagnosis related costs

**Fig. 1:**
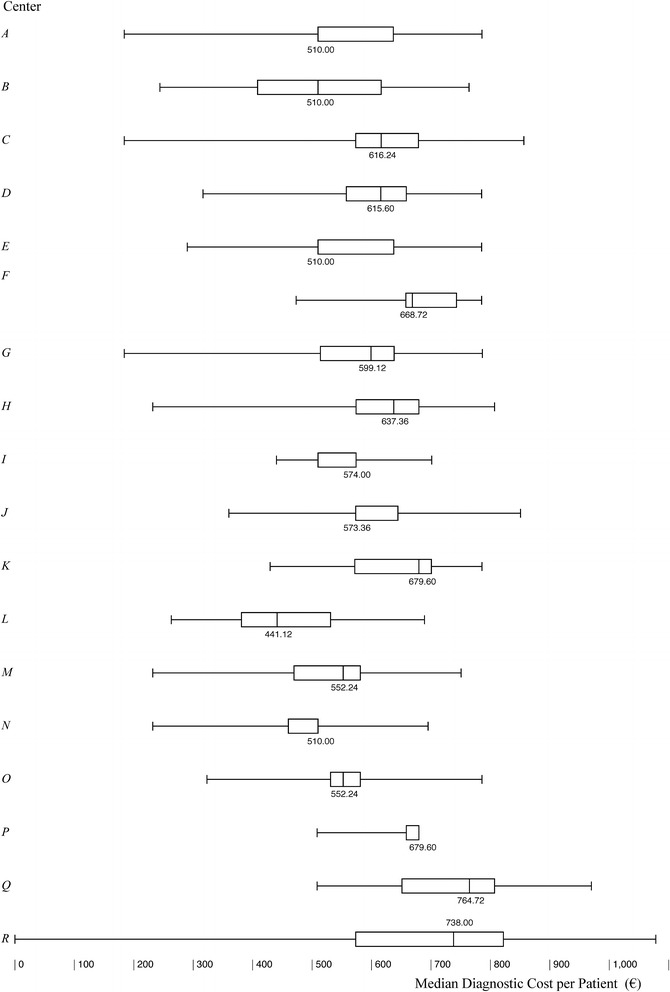
Total diagnostic cost (€) per patient: intercenter variability

Total diagnostic cost per patient to complete the diagnostic evaluation amounted to €574.00, of which €510.00 was related to recommended procedures and tests and €105.60 to optional examinations (Fig. 1; Additional file 1: Table 4). The multivariate analysis highlighted the following cost drivers: ADHD centre, sender, and time to diagnosis (Table 2). Concomitant psychiatric disorders, as well as other clinical and anamnestic variables, didn’t affect diagnosis costs (Table 2).

**Table 2:** Drivers of the diagnostic costs: multivariate analyses

Constant	Coefficient	CI 95%	p*
	378.55	(348.43–408.66)	<0.0001
Center			
A	66.24	(48.05–84.42)	<0.0001
B	20.25	(2.42–38.08)	0.0260
C	77.75	(59.81–95.69)	<0.0001
D	51.21	(30.99–71.42)	<0.0001
E	70.33	(48.53–92.12)	<0.0001
F	79.40	(58.18–100.62)	<0.0001
G	75.76	(55.44–96.108)	<0.0001
H	72.11	(54.92–89.31)	<0.0001
I	79.72	(58.63–100.8)	<0.0001
J	93.36	(75.5–111.22)	<0.0001
K	38.67	(15.63–61.71)	0.0010
L	–		
M	87.82	(13.78–161.86)	0.0201
N	46.85	(25.86–67.84)	<0.0001
O	84.22	(67.35–101.09)	<0.0001
P	82.20	(61.25–103.15)	<0.0001
Q	59.44	(32.61–86.28)	<0.0001
R	77.65	(56.14–99.15)	<0.0001
Gender			
Female	4.41	(−2.79 to 11.61)	ns
Male	–		
Age at diagnosis			
Years	0.70	(−1.14 to 2.54)	ns
Scholarship			
Primary	6.71	(−3.66 to 17.08)	ns
Middle	–		
Scholar support			
Yes	–		
None	9.56	(−0.84 to 19.97)	ns
Sender			
GP	8.70	(−5.78 to 23.19)	ns
Relatives	–		
School	9.47	(1.52–17.43)	0.0196
Neuropsychiatric (private)	5.38	(−5.89 to 16.65)	ns
Neuropsychiatric (NHS)	5.65	(−8.08 to 19.38)	ns
CANS	5.11	(−3.77 to 14)	ns
Familiar history of ADHD			
Positive	2.10	(−4.99 to 9.18)	ns
Negative	–		
Diagnosis of ADHD			
Yes	2.70	(−3.55 to 8.94)	ns
None	–		
Psychiatric concomitant disorders			
Yes	–		
None	0.66	(−5.02 to 6.33)	ns
CGIS			
<5	3.74	(−3.15 to 10.64)	ns
≥5	–		
Time to diagnosis			
Working days	0.05	(0.02–0.09)	0.0033

* Multivariate linear regression model

Statistically significant (Kruskal–Wallis, p < 0.001) inter-centre variability was related to the completion rate of the recommended set of assessments (Additional file 1: Table S5). The total cost of the diagnosis also varied in relation to the sender and was highest for patients referred from CANPS (€615.60). However, the relative increase was only 7.4% over the total median cost (Additional file 1: Table S6).

Globally, it took 119 days to complete the diagnostic pathway, with a wide variability mainly due to the centres (range from 51 to 302 days; Kruskal–Wallis, p < 0.0001) and the senders, with median time-to-diagnosis markedly reduced when the patient was referred by CANPS (91 days) or increased if referred by GPs (162 days), or by other specialist neuropsychiatrists practicing in agreement with the NHS (169.5 days; Kruskal–Wallis, p < 0.0001; Additional file 1: Table S7.

Whether or not the diagnosis of ADHD was confirmed on elapsed time was not statistical significative: 123 days if positive compared to 108 days if negative (Wilcoxon–Mann–Whitney, p = 0.0871). However, the time to diagnosis was slightly greater for patients receiving all the recommended tests (122 working days) compared to those who did not complete the assessment (111.5 days; Wilcoxon–Mann–Whitney, p = 0.0086).

**Fig. 2:**
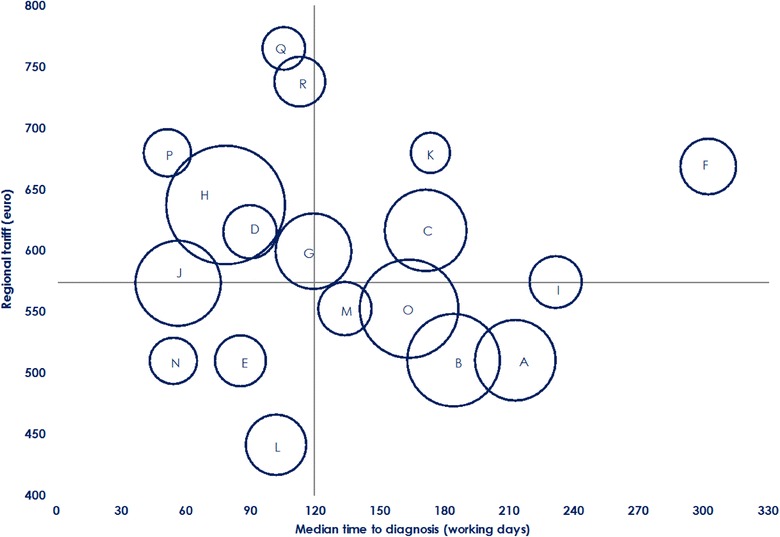
Median time to diagnosis (working days) and diagnostic costs (€): Intercenter variability. Bubbles’ size represents the number of diagnoses per center

**Fig. 3:**
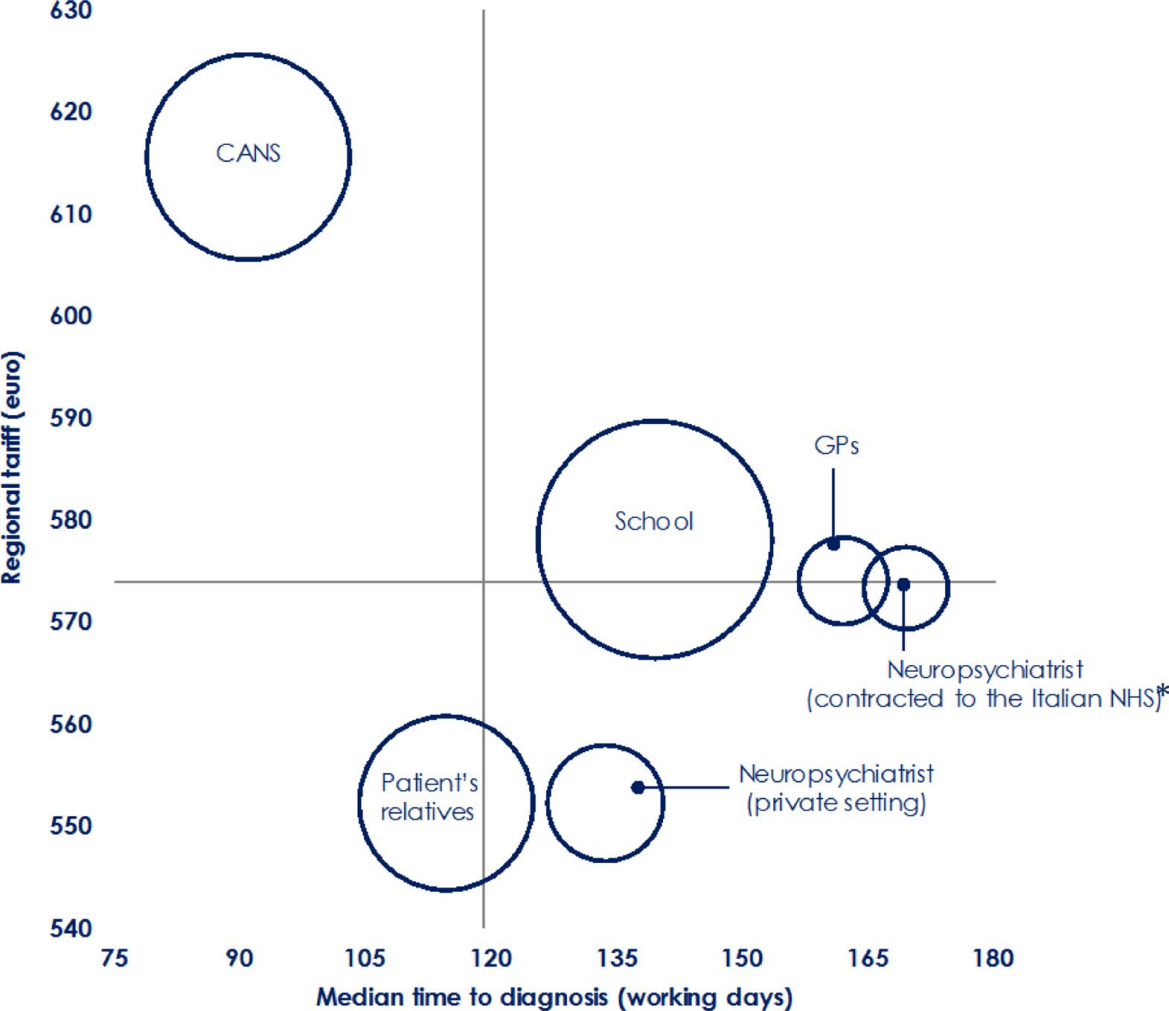
Median time to diagnosis (working days) and diagnostic costs (€): Variability by types of the senders. Bubbles’ size represents the number of diagnoses per sender; *CANS* Child and Adolescent Neuropsychiatry Service, *GP* General practitioner. *Asterisk* specialist neuropsychiatrists practicing in agreement with the NHS

Assuming that the time to diagnosis could be considered as a measure of efficiency by the regional health service (and by patients, too), Fig. 2 shows the wide variability among centres of the ratio of costs reimbursed by the health service and median time to diagnosis. This ratio varies significantly also in relation to the sending unit (Fig. 3).

### Treatment costs

The following annual treatment costs were assessed for patients with an ADHD diagnosis (n = 753):

- Non-pharmacological interventions: 1092 patients (86%), of whom 903 received a non-pharmacological intervention only (71%) and 189 (15%) combined with drugs;
- Drug treatments: 199 patients (16%), of whom 10 (1%) received drug treatment alone and 189 (90%) combined with non-pharmacological treatments.

The remaining 174 patients (14%) were not included because they were still being monitored (watchful-waiting) at the time of data extraction. Detailed information is presented in Additional file.

The median total treatment cost per patient, including laboratory and instrumental assessment needed to begin the drug therapy, was €830.00 (Additional file 1: Table S8), and resulted due mainly to the non-pharmacological therapy cost per patient. In addition to marked variability among centres (Kruskal–Wallis, p < 0.0001; Additional file 1: Table S9), non-pharmacological therapy resulted as being more expensive for patients concomitantly treated with drugs (€929) compared to those treated with psychological interventions alone (€590; Wilcoxon–Mann–Whitney p = 0.0064).

**Fig. 4:**
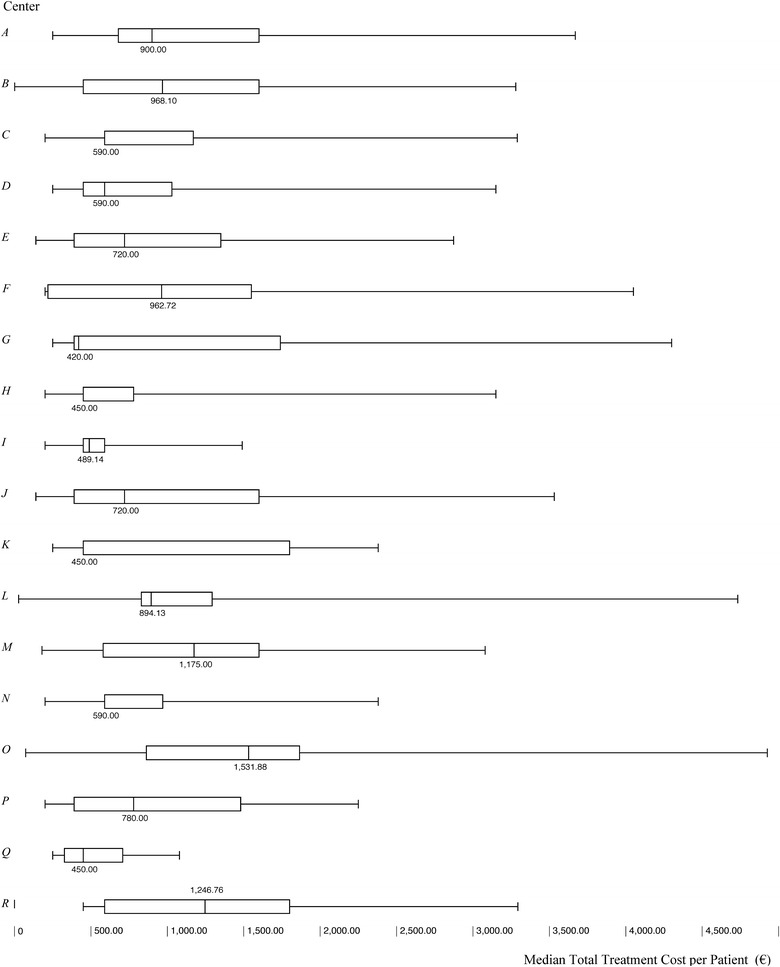
Total treatment cost (€) per patient: intercenter variability

Pharmacological treatments were prescribed in 14 out of 18 centres. Methylphenidate was the most used drug, prescribed in 170 patients (85.4%), followed by atomoxetine (10.1%). Median drug cost for 1 year was €97.60, of which €65.36 covered by stimulant treatment and €32.34 by non-stimulant treatment. Inter-centre variability (Fig. 4) was not statistically significant (Additional file 1: Table S10).

A total of 331 adverse events associated with drug treatments were reported, of which 9 (3%), 99 (30%), and 222 (67%) were classified as severe, moderate and mild, respectively. No action was required for 208 adverse events, while, for the remaining events, patients recovered upon drug discontinuation (n = 77, 23%) or dose changing (n = 46, 14%). No patient was treated or hospitalised due to adverse events so no adjunctive cost estimation was needed.

## Discussion

This study presents the first, and most comprehensive, estimate to date of the costs, from the NHS perspective, associated with both the diagnostic assessment and a 12-month therapy in children and adolescents with ADHD in Italy. The overall diagnostic and treatment costs per patient amounts to €574 and €830, respectively (median total: €1404). In our opinion, this is a reliable and representative estimate of ADHD health costs in Italy. Indeed, in our country, the management of ADHD patients was provided mainly by a network of specialized hubs on ADHD (Tier 3)—the Regional ADHD centres—who are responsible for following the most appropriate diagnostic procedures and treatment prescriptions according to the Italian guidelines [22, 43]. This process, in the Lombardy Region, was strictly monitored by the official Regional ADHD registry [40, 41] thus we can therefore expect that it accurately represents a cost estimate that is consistent with that of the management of ADHD in real clinical practice. The evaluation of ADHD costs presented in this article was calculated through a retrospective analysis of data inputted in this registry. Moreover, neuropsychiatrists working at the ADHD centres are also the only clinicians who, according to existing Italian legislation, can prescribe drug therapies for ADHD. This, in turn, gave us the possibility of calculating a reliable estimate also of the total treatment costs.

These costs are consistent, in some cases, and not, in others, with those previously suggested in other countries [32, 35, 50–56]. Such variability in ADHD costs reported may be explained by the different methodologies, especially diagnostic procedures, and criteria used in the studies, as well as by the different settings considered, and, for some authors, also the different cultural approaches [17]. In particular, psychopharmacological treatment in Italian children and adolescents is not the norm, and prescription rates for mental disorders are relatively low. A recent Italian study [40] shows that only 16% of ADHD patients are treated pharmacologically, compared with higher rates reported in other countries, suggesting that also for ADHD, the cultural education and disposition, and the professional attitude of the majority of the child psychiatrists of the Lombardy Region’s mental health services, are more inclined toward behavioural treatments than the use of drugs. To date, a cost analysis on ADHD in Italy has not yet been performed and published. An abstract presented in a conference contribution shows similar costs compared to those estimated in the present study [25]. This study, however, was methodologically different and did not calculate the diagnostic versus the therapeutic costs separately, and this does not allow us to make a more critical comparison with our findings.

Our cost analysis is relevant from the perspective of the Italian NHS not only for ADHD [27]. Indeed, the ADHD centre, the sender, and the time to diagnosis, but not the ADHD diagnosis itself, constitute cost drivers. We can thus expect these drivers to be common to other mental health disorders. Moreover, assuming that the time to diagnosis is a moderator measure of care efficiency, and considering the wide inter-centre variability in both the relationship between cost and time to diagnosis, and between cost and diagnosis completeness according to the National recommended guidelines, these data could serve as a measure for monitoring and reassessing the accreditation over time of the 18 regional centres as specialized hubs on ADHD.

Interestingly, when community CANPS (Tier 2) was the sender, time to diagnosis markedly decreased (1 month), with a relative increase (7%) over the total cost. As such, the recommended pathway of care by the Italian National Institute of Health (*Istituto Superiore di Sanità*, ISS) [57] when a child has a suspected diagnosis of ADHD states that the paediatrician should refer the child to the CANPS, and that the CANPS, after a psychiatric screening assessment (if necessary) should refer the patient to the ADHD centre (Tier 3). Our findings confirm that this suggested model of transition of care is likely to be a positive, cost-effective pathway, given that it ensures a more prompt response to care needs in exchange for an acceptable increase of direct health costs.

There is public concern that the more rapid efficacy, in symptomatic terms, of pharmacological therapy, combined with its lower cost compared to non-pharmacological interventions, could favour an increased use of drugs alone for the ADHD management [58]. As previously reported [16, 39, 40] this study showed a significantly higher prescription of non-pharmacological treatments, thus confirming that this alarming risk is absolutely not present in the Italian context. Indeed, the majority of the children with ADHD were not currently receiving medication. This is due, in part, to an Italian tradition that drug treatment should be reserved for those with more severe symptoms and impairments [59]. To some extent, the modest incremental cost of the combination of drug and behavioural interventions, compared to behaviour therapies alone resulting in the present study, representing for more effective, versus less effective, management strategies, as widely suggested, should be considered in terms of the best choice for each patient.

Moreover, it has also become apparent from our analysis that the cost of the non-pharmacological therapies is higher whether these are combined with drug treatment. This finding is probably related to the fact that patients requiring a combined treatment more often present greater severity in terms of both symptomatology and functional impairment. We can thus expect that, for these patients, not only is a more intensive (in terms of frequency) psychoeducational approach needed, but there is also a need that this approach be carried out in several different settings in the children’s life [58].

Finally, the ADHD project of the Lombardy Region ensured that the diagnostic and therapeutic protocol followed by the ADHD centres, on which the cost analysis was based, was strictly monitored by the official registry, that it has been established according to the main recommendations of the national guidelines and that it is representative of the real clinical practice of an entire region. Indeed, the compliance to the shared diagnostic and therapeutic evaluation, according to the project guidelines, estimated by the analysis of data recorded by all 18 ADHD centers is very high and homogeneous in the Regional context (total completeness: 93.6%; range: 81.7–99.1%). We can’t expect similar conclusions assuming to analyze ADHD health care differences and similarities between the Italian regions: various socioeconomic and service organization characteristics, i.e., may be explain a part of these differences and highlighting an important and broader issue, but not specific to the ADHD management. Limited literature data available from the Italian context are not enough to reach useful comparisons and comments about regional differences among ADHD management.

However, the Regional ADHD Registry, as the main tool to monitor the ADHD project, was designed as a disease-oriented registry collecting information not only on ADHD patients treated with pharmacological therapy (as provided by the National Registry) but also on all patients who access ADHD centers for a diagnosis of suspected ADHD.

These reasons strengthen the potential use of our findings as a proxy model to estimate the cost of the implementation of the guidelines in clinical practice for the development of similar projects in other Italian regions or for other mental health disorders.

### Limitations

A few study limitations should be mentioned. First, our estimate was based on tariffs reimbursed by the Regional Health Service that does reflect the direct medical costs and not all costs of care provided. Furthermore, the estimates did not include other perspectives considered in cost studies, such as societal and caregiver perspectives. Subjects with mental health problems, including ADHD patients, require support from several dimensions in life, not only from the healthcare system, i.e. social care, housing, and employment [27, 60]. Service utilization, outpatient care, and medications, however, are described as the main components of the economic impact of a disorder in mental health [3]. Second, although the Lombardy Region is the most populated region in Italy, representing about 17% of the national health care costs, all data originated from a single region of Italy, and this may affect the generalizability and comparability of the reported findings. However, studies evaluating costs of mental health problems are not easily generalisable from one country to another because service systems, funding arrangements, and relative prices can vary considerably. Third, the clinical effect of the treatments was not explored. It was therefore not possible to perform a cost-effectiveness evaluation, although, to our knowledge, this is the first study that estimates the ADHD costs for both diagnosis and therapeutic pathways, with previous studies typically focusing only on economic evaluation of the treatment.

### Implications for behavioral health

This study gives a reliable indication of the economic effect of both diagnosis and treatment of ADHD in Italy from the NHS perspective. There is clearly a need, however, for a comprehensive picture of the total health and societal costs of ADHD. There also is an urgent need for studies on cost-effectiveness of interventions and for consequent support arrangements for specialised ADHD services that address the needs of patients and their families so as geographically equitable and efficient as to the best evidence care management.

The costs associated with mental disorders are difficult to estimate, but continuing efforts to do so increase available evidence as well as the understanding of the struggles of the individuals and families who need appropriate and adequate care.

## Additional file

**Additional file 1.** Additional tables.
