# Supplementary material for: Italian regional health service costs for diagnosis and 1-year treatment of ADHD in children and adolescents
Source: Int J Ment Health Syst. 2017 Apr 28;11:33. doi: 10.1186/s13033-017-0140-8 (PMC5410029; doi:10.1186/s13033-017-0140-8)
Supplement: Supplementary file 1 — Additional file 1. Additional tables. [file 13033_2017_140_MOESM1_ESM.docx]

**Supplementary material**

**Table S1**. Regional tariffs of psychological diagnostic tests (Lombardy region, 2014)

| **Test** | | **Regional Tariff** | |
| --- | --- | --- | --- |
|  |  | **Code** | **Unit cost** |
| **Recommended** | Clinical interview | CCA010 | € 64.0 |
|  | Neurological examination | CA009 | € 50.0 |
|  | Cognitive level (IQ) | CA001 | € 70.0 |
|  | KSADS-DAWBA | AA041 | € 58.0 |
|  | CPRS – CBLC | AA041 | € 58.0 |
|  | CTRS | AW161 | € 150.0 |
|  | CGAS - CGIS | AV371 | € 60.0 |
| **Facultative** | AC-MT | AF591 | € 42.2 |
|  | ADHD Rating Scale | AA041 | € 58.0 |
|  | ANT | AF591 | € 64.0 |
|  | *Attenzione uditiva* | AF591 | € 21.1 |
|  | BIA | AF591 | € 64.0 |
|  | Brown ADD Scale | CA009 | € 50.0 |
|  | *Campanelle* | AF591 | € 21.1 |
|  | CASS | CA009 | € 50.0 |
|  | CBCL-self version | CA009 | € 50.0 |
|  | CDI | CA009 | € 50.0 |
|  | CP-paper version | AF591 | € 42.2 |
|  | CPT-pc version | AF591 | € 42.2 |
|  | MASC | CA009 | € 50.0 |
|  | Movement ABC | AF601 | € 35.0 |
|  | *Prove 2.3.4 dislessia* | AF591 | € 21.1 |
|  | *Prove di lettura MT* | AF591 | € 64.0 |
|  | SAFA | CA009 | € 50.0 |
|  | SAFA-A | CA009 | € 50.0 |
|  | SAFA-D | CA009 | € 50.0 |
|  | *Attenzione e concentrazione* | AF591 | € 42.2 |
|  | Corsi block test | AF591 | € 42.2 |
|  | Rey test | AF591 | € 42.2 |
|  | Tower of London test | AF591 | € 42.2 |
|  | TPV | AF591 | € 64.0 |
|  | VMI | AF591 | € 21.1 |
|  | WCST | AF591 | € 64.0 |

*Note. KSADS: Kiddie Schedule for Affective Disorders and Schizophrenia; DAWBA: Development and Well-Being Assessment; CPRS: Conners’ Parent Rating Scale; CBCL: child behavior checklist; CTRS: Conners’ Teacher Rating Scale; CGAS: Children's Global Assessment Scale; CGIS: Clinical Global Impressions - Severity scale; AC-MT: *Test di Valutazione delle Abilità di Calcolo e Problem Solving* – Italian assessment test for computing skills and problem solving; ANT: Attention Network Test; Attenzione uditiva: auditory attention test from the Italian battery of tests for ADHD; BIA: Italian battery of tests for ADHD; Brown ADD Scale: Brown Attention-Deficit Disorder Scales; Campanelle: *Test dell campanelle* – Italian bells test; CASS: Conners-Wells Adolescent Self-Report Scale; CDI: Children's Depression Inventory; CP cartaceo: continuous performance test – paper version; CPT informatico: continuous performance test – pc version; MASC: Multidimensional Anxiety Scale for Children; Movement ABC: Movement Assessment Battery for Children; Prove 2, 3 e 4 dislessia: items 2, 3, and 4 from the Italian battery for the assessment of dyslexia and developmental dysorthography; Prove di lettura MT: Prove di Lettura MT: Italian reading tests MT; SAFA: *Scale Psichiatriche di Autosomministrazione per Fanciulli e Adolescenti* - Italian psychiatric self-scales for children and adolescents; SAFA-A: Italian psychiatric self-scales for children and adolescents-anxiety; SAFA-D: Italian psychiatric self-scales for children and adolescents-depression; Attenzione e concentrazione: Italian attention and concentration tests; TPV: Developmental Test of Visual Perception; VMI: Developmental Test of Visual-Motor Integration; WCST: Wisconsin Card Sorting Test.

**Table S2**. Estimated cost of non-pharmacological therapies (region Lombardy, 2014)

| Therapy | Regional tariff | | Per patient | |
| --- | --- | --- | --- | --- |
|  | Code | Unit cost | Units | Cost |
| Parent training | AK111 | € 25.0 | 10 | € 250.0 |
| Teacher training | AN531 | € 20.0 | 7 | € 140.0 |
| Child training | AN411 | € 20.0 | 10 | € 200.0 |
| Cognitive | AL471 | € 20.0 | 45 | € 900.0 |
| Counselling | AA041 | € 58.0 | 10 | € 580.0 |
| Speech | AL461 | € 30.0 | 45 | € 1,350.0 |
| Psychodynamic | CA014 | € 60.0 | 45 | € 2,700.0 |
| Psychomotor | AL481 | € 30.0 | 45 | € 1,350.0 |
| Family | CA015 | € 100.0 | 10 | € 1,000.0 |

**Table S3**. Cost of pharmacological therapies

|  |  |  |  | Pack | | | | | NHS cost | | |  |
| --- | --- | --- | --- | --- | --- | --- | --- | --- | --- | --- | --- | --- |
| ATC | Drug | Branded/ Generic | Reimbursement^a^ | Form | Units | mg/unit | PP^b^ | MSP^c^ | | €/pack^d^ | €/mg | |
| C07AA | Propranolol | Branded | A | Tabs | 30 | 40 | 1.86 | 1.13 | | 1.86 | 0.0016 | |
| N03AG | Sodium valproate | Branded | A | Tabs | 40 | 500 | 7.89 | 4.78 | | 7.89 | 0.0004 | |
| N03AG | Sodium valproate | Branded | A | Tabs ER | 30 | 500 | 12.57 | 7.62 | | 12.57 | 0.0008 | |
| N05AA | Levopromazine | Branded | A | Tabs | 20 | 25 | 2.58 | 1.56 | | 2.58 | 0.0052 | |
| N05AD | Haloperidol | Branded | A | Drops | 15 | 10 | 3.45 | 2.09 | | 3.45 | 0.0230 | |
| N05AE | Ziprasidone | Generic | A | tabs | 56 | 40 | 62.22 | 37.70 | | 62.22 | 0.0278 | |
| N05AG | Pimozide | Branded | A | Tabs | 20 | 4 | 5.46 | 3.31 | | 5.46 | 0.0683 | |
| N05AX | Risperidone | Branded | A PHT | Drops | 100 | 1 | 37.96 | 23.00 | | 26.87 | 0.2687 | |
| N05AX | Risperidone | Generic | A | Tabs | 60 | 1 | 14.63 | 8.86 | | 14.63 | 0.2438 | |
| N05AX | Aripiprazole | Branded | A PHT | Tabs | 28 | 10 | 140.49 | 85.12 | | 99.45 | 0.3552 | |
| N06AB | Fluoxetine | Branded | A PHT | Drops | 240 | 10 | 10.71 | 6.49 | | 7.58 | 0.0032 | |
| N06AB | Fluvoxamine | Generic | A | Tabs | 30 | 50 | 7.94 | 4.81 | | 7.94 | 0.0053 | |
| N06AB | Sertraline | Generic | A | Tabs | 30 | 50 | 6.00 | 3.64 | | 6.00 | 0.0040 | |
| N06AB | Sertraline | Branded | A | Syrup | 60 | 20 | 19.83 | 12.02 | | 19.83 | 0.0165 | |
| N06BA | Methylphenidate | Branded | A PHT | Tabs ER | 30 | 10 | 19.94 | 12.08 | | 14.12 | 0.0471 | |
| N06BA | Methylphenidate | Branded | A PHT | Tabs | 30 | 10 | 5.96 | 3.61 | | 4.22 | 0.0141 | |
| N06BA | Atomoxetine | Branded | A PHT | Tabs | 28 | 60 | 116.77 | 70.75 | | 82.66 | 0.0492 | |

Note: ^a^ A: fully reimbursed in retail pharmacies; A PHT: 100% reimbursed under direct distribution; ^b^ Price to Public; ^c^ Manufacturer Selling Price; ^d^ PP of class A) medicine, or sum of MSP and 10.2% distribution margin on PP of class A-PHT medicine.

**Table S4**. Total diagnostic costs (€) per patient per center

| **Center** | **n** | **Completed recommended pathway** | | | **Diagnostic cost** | | **Facultative tests**  **Diagnostic costs** | | | | **Total diagnostic cost** | |
| --- | --- | --- | --- | --- | --- | --- | --- | --- | --- | --- | --- | --- |
|  |  | **No** | **Yes** | **% Yes** | **Median** | **IQR** | **n** | **%** | **Median** | **IQR** | **Median** | **IQR** |
| A | 140 | 55 | 85 | *61* | 510.0 | 58.0 | 63 | *45* | 0 | 127.4 | 510.0 | 107.6 |
| B | 185 | 87 | 98 | *53* | 510.0 | 150.0 | 112 | *61* | 64.0 | 127.4 | 510.0 | 208.0 |
| C | 144 | 13 | 131 | *91* | 510.0 | 0 | 119 | *83* | 126.7 | 84.5 | 616.2 | 116.2 |
| D | 64 | 20 | 44 | *69* | 510.0 | 65.0 | 64 | *100* | 126.7 | 63.4 | 615.6 | 106.6 |
| E | 57 | 13 | 44 | *77* | 510.0 | 0 | 30 | *53* | 42.2 | 148.5 | 510.0 | 127.4 |
| F | 70 | 1 | 69 | *99* | 510.0 | 0 | 70 | *100* | 158.7 | 85.1 | 668.7 | 85.1 |
| G | 126 | 76 | 50 | *40* | 450.0 | 60.0 | 124 | *98* | 128.0 | 85.1 | 599.1 | 124.0 |
| H | 297 | 44 | 253 | *85* | 510.0 | 0 | 275 | *93* | 127.4 | 105.6 | 637.4 | 105.6 |
| I | 61 | 7 | 54 | *89* | 510.0 | 0 | 44 | *72* | 64.0 | 64.0 | 574.0 | 64.0 |
| J | 155 | 4 | 151 | *97* | 510.0 | 0 | 146 | *94* | 63.4 | 71.1 | 573.4 | 71.1 |
| K | 36 | 11 | 25 | *69* | 510.0 | 150.0 | 36 | *100* | 169.6 | 53.4 | 679.6 | 128.9 |
| L | 82 | 57 | 25 | *30* | 360.0 | 150.0 | 75 | *91* | 21.1 | 100.0 | 441.1 | 150.0 |
| M | 64 | 26 | 38 | *59* | 510.0 | 150.0 | 52 | *81* | 63.4 | 63.4 | 552.2 | 109.7 |
| N | 50 | 14 | 36 | *72* | 510.0 | 150.0 | 12 | *24* | 0 | 0 | 510.0 | 50.0 |
| O | 208 | 11 | 197 | *95* | 510.0 | 0 | 193 | *93* | 42.2 | 50.0 | 552.2 | 45.4 |
| P | 51 | 5 | 46 | *90* | 510.0 | 0 | 50 | *98* | 169.6 | 0 | 679.6 | 21.1 |
| Q | 41 | 7 | 34 | *83* | 510.0 | 0 | 41 | *100* | 262.5 | 106.2 | 764.7 | 155.7 |
| R | 56 | 9 | 47 | *84* | 510.0 | 0 | 44 | *79* | 230.5 | 258.4 | 738.0 | 280.2 |
| ***All centers*** | ***1,887*** | ***460*** | ***1427*** | ***76*** | ***510.0*** | ***0*** | ***1,550*** | ***82*** | ***105.6*** | ***147.9*** | ***574.0*** | ***148.5*** |

**Table S5**. Completion rate of the recommended set of assessments for ADHD diagnosis

| **Recommended assessments** | **Facultative assessments** | **n** | **%** | **Median** | **IQR** | **Kruskall-Wallis test** |
| --- | --- | --- | --- | --- | --- | --- |
| Notcompleted | None | 124 | 7 | 360.0 | 33.0 | p<0.0001 |
| Notcompleted | Done | 336 | 18 | 514.0 | 141.6 |  |
| Completed | None | 213 | 11 | 510.0 | 0 |  |
| Completed | Done | 1,214 | 64 | 637.4 | 105.6 |  |

**Table S6**. Median diagnostic cost (€) per patient per sender (overall median cost: €570.00)

| **Sender** | **n** | **Median** | **Kruskall-Wallis test** |
| --- | --- | --- | --- |
| School | 648 | 578.0 | p<0.0001 |
| CANS | 485 | 615.6 |  |
| Relatives | 356 | 552.2 |  |
| Neuropsychiatrist (private setting) | 164 | 552.2 |  |
| GP | 95 | 574.0 |  |
| Neuropsychiatrist (contracted to the Italian NSH) | 86 | 573.4 |  |

**Table S7**. Median time to diagnosis (working days)

| **Center** | **n** | **Q1** | **Median** | **Q3** | **IQR** | **Kruskall-Wallis test** |
| --- | --- | --- | --- | --- | --- | --- |
| A | 140 | 143.5 | 212.5 | 260.5 | 117.0 | p<0.0001 |
| B | 185 | 80.0 | 184.0 | 267.0 | 187.0 |  |
| C | 144 | 122.0 | 171.0 | 222.0 | 100.0 |  |
| D | 64 | 58.0 | 89.5 | 137.5 | 79.5 |  |
| E | 57 | 63.0 | 85.0 | 132.0 | 69.0 |  |
| F | 70 | 261.0 | 302.0 | 388.0 | 127.0 |  |
| G | 126 | 85.0 | 119.0 | 164.0 | 79.0 |  |
| H | 297 | 59.0 | 78.0 | 117.0 | 58.0 |  |
| I | 61 | 163.0 | 231.0 | 288.0 | 125.0 |  |
| J | 155 | 33.0 | 56.0 | 101.0 | 68.0 |  |
| K | 36 | 130.5 | 173.0 | 225.0 | 94.5 |  |
| L | 82 | 70.0 | 101.5 | 121.0 | 51.0 |  |
| M | 64 | 96.0 | 133.5 | 205.5 | 109.5 |  |
| N | 50 | 30.0 | 54.0 | 87.0 | 57.0 |  |
| O | 208 | 104.0 | 163.0 | 222.0 | 118.0 |  |
| P | 51 | 43.0 | 51.0 | 77.0 | 34.0 |  |
| Q | 41 | 71.0 | 105.0 | 122.0 | 51.0 |  |
| R | 56 | 61.5 | 112.5 | 185.0 | 123.5 |  |
| ***All centers*** | ***1.887*** | ***69.0*** | ***119.0*** | ***204.0*** | ***135.0*** |  |

**Table S8**. Median total treatment costs (€) per patient: intercenter variability

| **Center** | **N** | **Q1** | **Median** | **Q3** | **IQR** | **Kruskall-Wallis test** |
| --- | --- | --- | --- | --- | --- | --- |
| A | 29 | 830.0 | 1,030.0 | 1,600.0 | 770.0 | p <0.0001 |
| B | 150 | 450.0 | 1,075.8 | 1,600.0 | 1,150.0 |  |
| C | 69 | 590.0 | 590.0 | 1,170.0 | 580.0 |  |
| D | 32 | 450.0 | 590.0 | 1,030.0 | 580.0 |  |
| E | 31 | 390.0 | 780.0 | 1,350.0 | 960.0 |  |
| F | 48 | 450.0 | 1,126.8 | 1,614.1 | 1,164.1 |  |
| G | 106 | 390.0 | 420.0 | 1,740.0 | 1,350.0 |  |
| H | 119 | 450.0 | 450.0 | 830.0 | 380.0 |  |
| I | 49 | 450.0 | 520.0 | 590.0 | 140.0 |  |
| J | 87 | 390.0 | 720.0 | 1,600.0 | 1,210.0 |  |
| K | 35 | 450.0 | 450.0 | 1,800.0 | 1,350.0 |  |
| L | 59 | 830.0 | 1,120.9 | 1,525.1 | 695.1 |  |
| M | 38 | 633.3 | 1,175.0 | 1,600.0 | 966.7 |  |
| N | 29 | 590.0 | 590.0 | 970.0 | 380.0 |  |
| O | 148 | 1,030.0 | 1,630.0 | 2,043.6 | 1,013.6 |  |
| P | 19 | 390.0 | 780.0 | 1,480.0 | 1,090.0 |  |
| Q | 11 | 450.0 | 558.6 | 939.9 | 489.6 |  |
| R | 43 | 590.0 | 1,478.8 | 1,894.7 | 1,304.7 |  |
| ***All centers*** | ***1,102*** | ***450.0*** | ***830.0*** | ***1,600.0*** | ***1,150.0*** |  |

*Note: 174 patients under monitoring (watchful waiting) were excluded. Total treatment costs include non-pharmacological therapies, pharmacological treatments and pre-drug testing.

**Table S9**. Median non-pharmacological therapy costs (€) per patient: intercenter variability

| **Center** | **n** | **Q1** | **Median** | **Q3** | **IQR** | **Kruskall-Wallis test** |
| --- | --- | --- | --- | --- | --- | --- |
| A | 29 | 580.0 | 830.0 | 1.480.0 | 900.0 | p<0.0001 |
| B | 147 | 250.0 | 900.0 | 1.450.0 | 1.200.0 |  |
| C | 69 | 590.0 | 590.0 | 1.170.0 | 580.0 |  |
| D | 32 | 420.0 | 590.0 | 1.030.0 | 610.0 |  |
| E | 31 | 390.0 | 720.0 | 1.350.0 | 960.0 |  |
| F | 48 | 200.0 | 900.0 | 1.550.0 | 1.350.0 |  |
| G | 106 | 390.0 | 420.0 | 1.740.0 | 1.350.0 |  |
| H | 118 | 450.0 | 450.0 | 780.0 | 330.0 |  |
| I | 49 | 450.0 | 450.0 | 590.0 | 140.0 |  |
| J | 87 | 390.0 | 720.0 | 1.600.0 | 1.210.0 |  |
| K | 35 | 450.0 | 450.0 | 1.800.0 | 1.350.0 |  |
| L | 57 | 830.0 | 830.0 | 970.0 | 140.0 |  |
| M | 37 | 580.0 | 1.150.0 | 1.550.0 | 970.0 |  |
| N | 29 | 590.0 | 590.0 | 970.0 | 380.0 |  |
| O | 148 | 830.0 | 1.480.0 | 1.730.0 | 900.0 |  |
| P | 19 | 390.0 | 780.0 | 1.480.0 | 1.090.0 |  |
| Q | 8 | 450.0 | 450.0 | 680.0 | 230.0 |  |
| R | 43 | 590.0 | 1.150.0 | 1.600.0 | 1.010.0 |  |
| ***All centers*** | ***1.092*** | ***450.0*** | ***830.0*** | ***1.520.0*** | ***1.070.0*** |  |

*Note: 174 patients under monitoring (watchful waiting) were excluded.

**Table S10**. Median drug treatment costs (€) per patient: intercenter variability

| **Center** | **n** | **Q1** | **Median** | **Q3** | **IQR** | **Kruskall-Wallis test** |
| --- | --- | --- | --- | --- | --- | --- |
| A | 8 | 96.2 | 186.6 | 307.8 | 211.5 | p=0.0579 |
| B | 52 | 46.0 | 100.9 | 280.8 | 234.8 |  |
| C | 7 | 65.1 | 93.5 | 150.7 | 85.7 |  |
| E | 6 | 33.3 | 49.2 | 56.6 | 23.3 |  |
| F | 7 | 21.7 | 33.5 | 138.7 | 117.0 |  |
| H | 2 | 655.2 | 741.9 | 828.6 | 173.4 |  |
| I | 1 | 78.3 | 78.3 | 78.3 | - |  |
| J | 4 | 23.1 | 38.0 | 66.0 | 42.9 |  |
| L | 38 | 51.3 | 74.6 | 170.6 | 119.3 |  |
| M | 3 | 151.3 | 179.6 | 226.3 | 75.0 |  |
| O | 42 | 52.0 | 81.0 | 199.0 | 147.1 |  |
| P | 2 | 40.1 | 70.9 | 101.7 | 61.6 |  |
| Q | 6 | 51.3 | 281.4 | 326.6 | 275.2 |  |
| R | 21 | 85.2 | 101.5 | 427.6 | 342.4 |  |
| ***All centers*** | ***199*** | ***49.4*** | ***97.6*** | ***241.6*** | ***192.2*** |  |
